# Alkaloids Isolated from the Lateral Root of *Aconitum carmichaelii*

**DOI:** 10.3390/molecules17089939

**Published:** 2012-08-20

**Authors:** Liang Xiong, Cheng Peng, Xiao-Fang Xie, Li Guo, Cheng-Jun He, Zhao Geng, Feng Wan, Ou Dai, Qin-Mei Zhou

**Affiliations:** 1 State Key Laboratory Breeding Base of Systematic Research Development and Utilization of Chinese Medicine Resources, Sichuan Province and Ministry of Science and Technology, Chengdu 610075, Sichuan, China; Email: xiling0505@126.com (L.X.); xxf14544@163.com (X.-F.X.); gli64@sina.com (L.G.); heye1217@163.com (C.-J.H.); gengzhao713@hotmail.com (Z.G.); wanfengcdzy@126.com (F.W.); haiou0505@126.com (O.D.); zhqmyx@sina.cn (Q.-M.Z.); 2 Key Laboratory of Standardization of Chinese Herbal Medicine, Ministry of Education, Chengdu 610075, Sichuan, China; 3 Pharmacy College, Chengdu University of Traditional Chinese Medicine, Chengdu 610075, Sichuan, China

**Keywords:** *Aconitum carmichaelii*, lateral root, alkaloids, bioactivities

## Abstract

Two new alkaloids, aconicarmine (**1**) and aconicaramide (**5**), were isolated from the EtOH extract of the lateral roots of *Aconitum carmichaelii*, together with five known compounds: fuziline (**2**), neoline (**3**), *N*-ethylhokbusine B (**4**), 5-hydroxymethylpyrrole-2-carbaldehyde (**6**), and oleracein E (**7**). Their structures were elucidated by physical and NMR analysis. Pyrrole alkaloids were isolated from *A. carmichaelii* for the first time. In the *in vitro* assays, compounds **2** and **3** showed activity against pentobarbital sodium-induced cardiomyocytes damage by obviously recovering beating rhythm and increasing the cell viability, while compounds **5** and **7** showed moderate antibacterial activity.

## 1. Introduction

*Aconitum carmichaelii* Debx. (Ranunculaceae) is widely distributed and cultivated in China's Sichuan province [1]. The parent and lateral roots of *A. carmichaelii*, two well known traditional Chinese medicines, named “chuan wu” and “fu zi” respectively in Chinese, have been widely used in China to treat various symptoms such as cadianeuria, neuralgia, rheumatalgia, and inflammation [1,2]. Previous chemical studies of this plant have led to the isolation of more than 66 diterpenoid alkaloids, four flavonoids, a ceramide, a steroid saponin, and a pyrimidine [2,3,4,5]. In addition, 147 diterpenoid alkaloids, including a lot of lipo-alkaloids, have been reported and identified by means of LC-MS analysis [6,7]. In searching for bioactive natural products from “fu zi”, two new alkaloids, aconicarmine (**1**) and aconicaramide (**5**), were isolated from the lateral roots, together with five known compounds: fuziline (**2**) [8], neoline (**3**) [9], *N*-ethylhokbusine B (**4**) [10], 5-hydroxymethyl-pyrrole-2-carbaldehyde (**6**) [11], and oleracein E (**7**) [12]. This paper describes the isolation, structure elucidation, and bioassays of these isolates.

## 2. Results and Discussion

**Figure 1:**
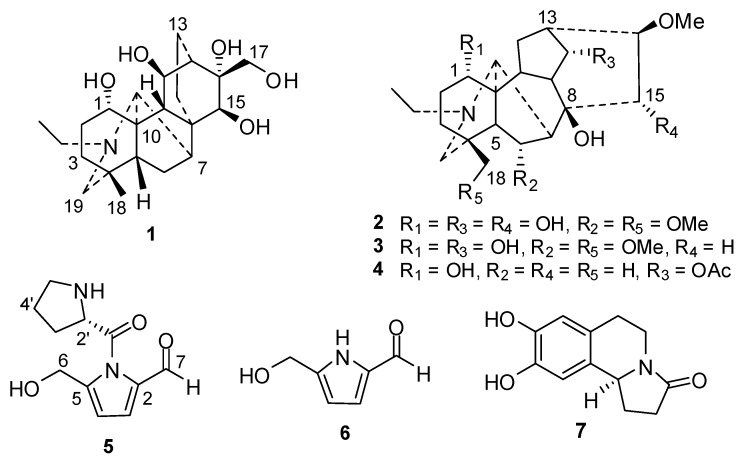
Structures of compounds **1**–**7**.

The EtOH extract of the lateral roots of *A. carmichaelii* was suspended in water and successively partitioned with petroleum ether, EtOAc, and *n*-BuOH. Separation of the *n*-BuOH fraction by column chromatography provided compounds **1**−**7** (Figure 1).

**Figure 2:**
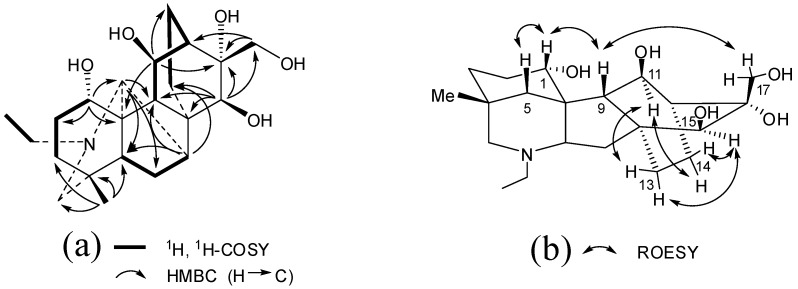
(**a**) Key ^1^H, ^1^H-COSY and HMBC correlations of aconicarmine(**1**); (**b**) Key ROESY correlations of aconicarmine(**1**).

**Figure 3:**
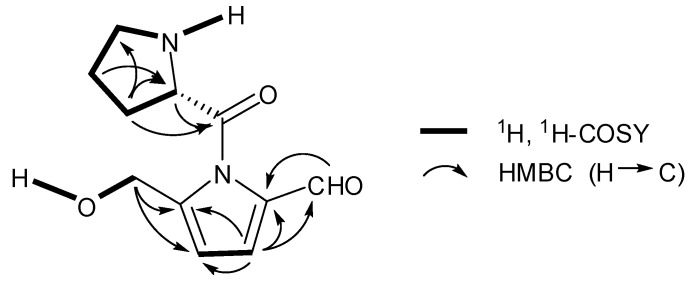
Key ^1^H, ^1^H-COSY and HMBC correlations of aconicaramide(**5**).

Compound **1** was obtained as colorless needles. The molecular formula C_22_H_35_NO_5_, with six degrees of unsaturation, was indicated by HR-ESI-MS *m/z* 394.2596 [M+H]^+^ (calcd for C_22_H_36_NO_5_, 394.2593) and NMR data (Table 1). The ^1^H-NMR spectrum displayed resonances assignable to an angular methyl group (*δ*_H_ 0.75, 3H, s, H-18), a N-ethyl group (*δ*_H_ 1.10, 3H, t, *J* = 7.2 Hz, H-22 and *δ*_H_ 2.48, 2H, *q*, *J* = 7.2 Hz, H-21), a *N*-methine group (*δ*_H_ 3.75, 1H, br s, H-20), three oxymethines (*δ*_H_ 3.83, 1H, s, H-15; *δ*_H_ 4.18, 1H, dd, *J* = 4.8, 10.2 Hz, H-1; and *δ*_H_ 4.56, 1H, d, *J* = 9.6 Hz, H-11), and an isolated oxymethylene group (*δ*_H_ 3.79, 1H, d, *J* = 12.0 Hz, H-17a and *δ*_H_ 4.03, 1H, d, *J* = 12.0 Hz, H-17b). In addition, it showed partially overlapped resonances ascribable to several aliphatic methylenes and methines between *δ*_H_ 0.99 and 2.86 ppm. The ^13^C-NMR and DEPT spectra of **1** revealed 22 carbon resonances corresponding to the above protonated units and four quaternary carbons (one oxygen-bearing, *δ*_C_ 80.3). The above-mentioned spectroscopic data suggested that **1** was a C_20_-diterpenoid alkaloid with an atisine-denudatine skeleton and an *N*-ethyl group [13]. Detailed comparison of the NMR data and the molecular composition of **1** with those of 11-*epi*-16*α*,17-dihydroxylepenine [14] indicated that compound **1** was an isomer of the latter. The ^13^C-NMR spectrum showed high similarity between them, except that the signal of C-11 (*δ*_C_ 72.3) in **1** was deshielded by 7.5 ppm compared to that of 11-*epi*-16*α*,17-dihydroxylepenine possessing an *α* hydroxy group at C-11. This revealed **1** was an 11*β*-hydroxyepimer [14], which was proved by 2D NMR experiments, including the ROESY analysis. The five OH groups could be located at C-1, C-11, C-15, C-16, and C-17, respectively, according to their HMBC correlations (Figure 2). In the ROESY spectrum, the correlations of H-11 and H-15 with H-13 and H-14, the same as those of lepenine [15], verified the OH-11 and OH-15 groups were *β*-oriented. Moreover, the correlations of H-1/H-5, H-1/H-9, and H-9/H-17 demonstrated the *α*-configuration of OH-1 and OH-16 (Figure 3). Accordingly, Compound **1** was established to be 16*α*,17-dihydroxylepenine and named aconicarmine.

**Table 1:** ^1^H- (400 MHz) and ^13^C-NMR (100 MHz) data of **1** (in CD_3_OD, *δ* in ppm, *J* in Hz).

No.	*δ*_H_	*δ*_C_	No.	*δ*_H_	*δ*_C_
1	4.18 dd (10.8, 6.4)	71.3	12	1.60 m	46.2
2	1.78 m, 2.40 m	31.8	13	1.42 m, 1.92 m	21.6
3	1.39 m, 1.61 m	39.7	14	1.03 m, 1.84 m	28.3
4	–	34.6	15	3.83 s	86.4
5	1.37 d (8.0)	54.3	16	–	80.3
6	1.26 dd (14.0, 5.2)	24.5	17	3.79 d (12.0)	69.2
	2.86 dd (14.0, 8.0)	–		4.03 d (12.0)	–
7	2.12 d (5.2)	43.5	18	0.75 s	26.4
8	–	44.9	19	2.31 d (11.2), 2.58 d (11.2)	58.1
9	1.82 d (9.6)	51.8	20	3.75 br s	68.5
10	–	52.1	21	2.48 m, 2.60 m	52.1
11	4.56 d (9.6)	72.3	22	1.10 t (7.2)	13.7

Compound **5**, obtained as a white powder, had the molecular formula C_11_H_14_N_2_O_3_ as indicated by HR-ESI-MS *m*/*z* 245.0906 [M+Na]^+^ (calcd for C_11_H_14_N_2_O_3_Na, 245.0902). The ^1^H-NMR spectrum of **5** displayed signals ascribed to an aldehyde group (*δ*_H_ 9.37, 1H, s, H-7), a pair of coupled olefinic methine protons (*δ*_H_ 6.19, 1H, d, *J* = 4.0 Hz, H-4 and *δ*_H_ 6.95, 1H, d, *J* = 4.0 Hz, H-3), an isolated oxymethylene group (*δ*_H_ 4.65, 2H, s, H-6), and an exchangeable proton (*δ*_H_ 4.39, s). These data, similar to those of **6** (Table 2), together with the carbon signals at (*δ*_C_ 178.8, 144.5, 133.4, 125.5, 110.0, and 56.7), indicated the presence of a pyrrole ring with the substitutions of an aldehyde and a hydroxymethyl group [16]. This was confirmed by HMBC correlations of H-3 with C-2, C-4, C-5, and C-7, H-6 with C-4 and C-5 (Figure 3).

**Table 2:** ^1^H- (400 MHz) and ^13^C-NMR (100 MHz) data of **5** and **6** (in CD_3_COCD_3_, *δ* in ppm, *J* in Hz).

No.	*δ*_H_			*δ*_C_	
	5	6		5	6
1	–	10.88 s		–	–
2	–	–		133.4	132.6
3	6.95 d (4.0)	6.91 d (4.0)		125.5	125.7
4	6.19 d (4.0)	6.22 d (4.0)		110.0	108.5
5	–	–		144.5	142.0
6	4.65 s	4.64 s		56.7	56.8
7	9.37 s	9.47 s		178.8	178.1
OH-6	4.39 s	4.29 s		–	
1′	6.69 ( *s*)			–	
2′	5.00 dd (10.8, 5.6)			57.5	
3′	1.97 m, 2.23 m			30.1	
4′	1.97 m, 2.29 m			23.6	
5′	3.31 m, 3.63 m			42.7	
6′	–			169.2	

Comparison of the NMR data between **5** and **6** indicated that they differed in the presence of resonances attributable to an additional prolyl moiety (*δ*_C_ 23.6, 29.9, 42.7, 57.5, 169.2) in **5** [17]. In addition, almost no downfield shift of H-6 was observed in **5** as compared with that of **6**, suggesting that the prolyl unit was attached to N instead of OH-6. This conjecture was refined by a ^1^H-^1^H COSY correlation observed between the exchangeable proton (*δ*_H_ 4.39) and H-6 (*δ*_H_ 4.65), as well as no HMBC correlation of H-6 with the carbonyl (C-6′).

Thus, the planar structure of **5** was established. The (*S*)-configuration at C-2′ was deduced by the negative specific rotation ([*α*]^20^_D_ = −75.0), consistent with that of (*S*)-proline [18], but opposite that of (*R*)-proline [19]. Therefore, compound **5** was determined as *N*-(L-prolyl)-5-hydroxymethyl-1*H*-pyrrole-2-carbaldehyde and named aconicaramide. 

The protective activities of the compounds against cardiomyocyte damage induced by pentobarbital sodium in primary cultured neonatal rat cardiomyocytes were investigated by the MTT method. The results showed that pentobarbital sodium induced a significant inhibition of MTT reduction. At concentrations of 10 μM, 1 μM, and 0.1 μM, compounds **2** and **3** increased the cell viability obviously (Table 3) and recovered beating rhythm when examined under a microscope. In addition, compound **5** showed moderate antibacterial activity against *Macrococcus caseolyticus*, *Staphylococcus epidermidis* and *Staphylococcus aureus* (MIC 200, 400 and 800 *μ*g/mL, respectively), while compound **7** displayed antibacterial activity against *Staphylococcus aureus*, *Macrococcus caseolyticus*, *Klebsiella pneumoniae* and *Streptococcus pneumoniae* (MIC 50, 200, 200 and 200 *μ*g/mL, respectively).

**Table 3:** Protective effects of **2** and **3** against cardiomyocyte damage induced by pentobarbital sodium.

Compound	Increase of the cell viability (%)		
	10 μM	1 μM	0.1 μM
**2**	65.44	64.14	63.09
**3**	73.82	72.51	47.64

## 3. Experimental

### 3.1. General

NMR spectra were recorded on a Bruker-AV-400 spectrometer. HRESIMS were measured with Waters Synapt G_2_ HDMS. IR were recorded on a Vector 22 FT-IR spectrometer. UV spectra were obtained on a Shimadzu UV-260 spectrophotometer. Optical rotations were measured with a Perkin-Elmer 341 plus. Column chromatography was performed with silica gel (200–300 mesh, Yantai Institute of Chemical Technology, Yantai, China), Al_2_O_3_ (100–200 mesh, Shanghai Ludu Chemical Reagent Factory, Shanghai 200000, China), MCI gel CHP 20P (75–150 *μ*m, Mitsubishi Chemical, Co., Japan), and Sephadex LH-20 (Amersham Pharmacia Biotech AB, Uppsala, Sweden).

### 3.2. Plant Material

The lateral root of *A. carmichaelii* was collected in July of 2010 from the culture field in Jiangyou, Sichuan postal code, China. Plant identity was verified by Prof. Min Li (Chengdu University of TCM, Sichuan, China). A voucher specimen (SFZ-0710) was deposited at the School of Pharmacy, Chengdu University of TCM, Chengdu, China.

### 3.3. Extraction and Isolation

The air-dried lateral roots (5 kg) of *A. carmichaelii* were extracted three times with 95% EtOH (30 L) for 2 h under reflux. The EtOH extract was concentrated *in vacuo* to yield a semi-solid (620 g), which was suspended in water and then extracted successively with petroleum ether, EtOAc and *n*-BuOH (5 × 2.5 L, 25 °C). The *n*-BuOH extract (85 g) was subjected to silica gel CC using a gradient elution of CHCl3–MeOH (50:1–1:1) to afford eleven fractions (Fractions A–K). Fraction B was further separated by Sephadex LH-20 (CHCl_3_–MeOH 1:1) to give five subfractions (B1–B5). The successive separation of B2 with Sephadex LH-20 (MeOH-H_2_O 1:1) and with PTLC (CHCl_3_-Me_2_CO 15:1) yielded **4** (5 mg), **5** (10 mg), and **6** (7 mg). B4 was fractioned by Sephadex LH-20 (petroleum ether−CHCl_3_−MeOH, 2:2:1) to give **7** (21 mg). Compound **3** (1.2 g) was crystallizated from Fraction D and then recrystallizated with CHCl_3_. Fraction G was separated by flash chromatography over MCI gel with a gradient of increasing MeOH (20%–100%) in water, to yield subfractions G1–G6. G2 was purified via Sephadex LH-20 (MeOH–H_2_O 1:1) followed by crystallization to yield **2** (0.9 g). Separation of G5 by chromatography over Al_2_O_3_ (CHCl_3_−MeOH, 2:1) and Sephadex LH-20 (MeOH–H_2_O 1:1) to afford **1** (170 mg).

*16α,17-Dihydroxylepenine* (**1**): Colorless needles, [*α*]^20^_D_ = −29.8 (*c* = 0.10, MeOH); IR (KBr) ν_max_: 3421, 2927, 1459, 1383, 1064 cm^−1^; ESI-MS *m/z* 394.2 [M+H]^+^, 416.2 [M+Na]^+^; HRESI-MS: *m/z* 394.2596 [M+H]^+^ (calcd for C_22_H_36_NO_5_, 394.2593); ^1^H- and ^13^C-NMR data see Table 1. 

*N-(L-Prolyl)-5-hydroxymethyl-1H-pyrrole-2-carbaldehyde* (**5**): White powder, [*α*]^20^_D_ = −75.0 (*c* = 0.28, MeOH); UV (MeOH) λ_max_: 202 (4.16), 258 (4.04), 294 (4.28) nm; IR (KBr) ν_max_: 3309, 2941, 2872, 1656, 1488, 1449, 1328, 1296, 1032, 776 cm^−1^; ESI-MS *m/z* 245.1 [M+Na]^+^; HRESI-MS *m/z* 245.0906 [M+Na]^+^ (calcd for C_11_H_14_N_2_O_3_Na, 245.0902); ^1^H- and ^13^C-NMR data see Table 2.

### 3.4. Cardiomyocyte Protection Assay

Neonatal rat cardiomyocytes were cultured in 96-well plates with DMEM media supplemented with 15% FBS. Cultures were maintained in a 37 °C humidified incubator with 5% CO_2_. On the fifth day when the cardiomyocytes were in the growth with rhythmical beating, they were exposed to the medium containing pentobarbital sodium at a concentration of 8 mg/mL. After 8 min, the medium was replaced with serum free medium including compounds at concentrations of 10 μM, 1 μM, and 0.1 μM, respectively, and incubated for 24 h. Then, 10 μL of MTT solution (5 mg/mL) was added and incubated for 4 h. Absorbance was measured at both 570 nm and 655 nm, and cell viability was evaluated with the deviations between them.

### 3.5. Antibacterial Activity Experiments

All bacteria were obtained from clinical samples and stored in the Department of Pharmacology of Chengdu University of TCM. The *in vitro* antibacterial activity was determined by the standard agar dilution method, according to NCCLS (National Committee for Clinical Laboratory Standard). 2 μL of cultures of test strains at the concentration of 1 × 10^6^ CFU/mL were inoculated on Mueller Hinton agar containing different concentrations of the test compounds. The MIC values were determined after incubation at 35–37 °C for 18–24 h.

## 4. Conclusions

Two new alkaloids aconicarmine (**1**) and aconicaramide (**5**) were isolated from the lateral roots of *A. carmichaelii*, together with five known alkaloids. Compounds **5** and **6** were the first report of pyrrole alkaloids from *A. carmichaelii*. Compounds **2** and **3** showed activity against pentobarbital sodium-induced cardiomyocytes damage by recovering beating rhythm and increasing the cell viability obviously. Compounds **5** and **7** showed moderate antibacterial activity.
